# Oriented Carbon Nanostructures from Plasma Reformed Resorcinol-Formaldehyde Polymer Gels for Gas Sensor Applications

**DOI:** 10.3390/nano10091704

**Published:** 2020-08-29

**Authors:** Neelakandan M. Santhosh, Aswathy Vasudevan, Andrea Jurov, Gregor Filipič, Janez Zavašnik, Uroš Cvelbar

**Affiliations:** 1 Department of Gaseous Electronics, Jožef Stefan Institute, Jamova cesta 39, SI-1000 Ljubljana, Slovenia; Neelakandan.M.Santhosh@ijs.si (N.M.S.); Aswathy.Vasudevan@ijs.si (A.V.); andrea.jurov@ijs.si (A.J.); gregor.filipic@ijs.si (G.F.); janez.zavasnik@ijs.si (J.Z.); 2 Jožef Stefan International Postgraduate School, Jamova cesta 39, SI-1000 Ljubljana, Slovenia

**Keywords:** inductively coupled plasma, oriented carbon nanostructures, plasma surface treatment, polymer gel, Raman spectroscopy

## Abstract

Oriented carbon nanostructures (OCNs) with dominant graphitic characteristics have attracted research interest for various applications due to the excellent electrical and optical properties owing to their vertical orientation, interconnected structures, electronic properties, and large surface area. Plasma enhanced chemical vapor deposition (PECVD) is considered as a promising method for the large-scale synthesis of OCNs. Alternatively, structural reformation of natural carbon precursor or phenol-based polymers using plasma-assisted surface treatment is also considered for the fabrication of OCNs. In this work, we have demonstrated a fast technique for the synthesis of OCNs by plasma-assisted structure reformation of resorcinol-formaldehyde (RF) polymer gels using radio-frequency inductively coupled plasma (rf-ICP). A thin layer of RF polymer gel cast on a glass substrate was used as the carbon source and treated with rf plasma under different plasma discharge conditions. Argon and hydrogen gases were used in surface treatment, and the growth of carbon nanostructures at different discharge parameters was systematically examined. This study explored the influence of the gas flow rate, the plasma power, and the treatment time on the structural reformation of polymer gel to produce OCNs. Moreover, the gas-sensing properties of as-prepared OCNs towards ethanol at atmospheric conditions were also investigated.

## 1. Introduction

Carbon nanostructures (CNs) have several unique structural, morphological, and electrical properties [1]. Owing to their structure, morphology, and preferential orientation, CNs have gained research attention in different fields of electronic, gas sensing, and energy storage applications. The structure, morphology, and orientation of CNs can be tuned by the parameters of the growth process. The orientation of surface-bound CNs can be arbitrary, horizontal, or vertical to the substrate. Among them, vertical OCNs are considered to be a promising material for the industrial and practical applications due to their high-aspect-ratio, large specific area, and good electrical properties that can improve the material performance. Commonly investigated forms of such CNs are vertical carbon nanotubes [2,3], vertical graphene sheets [4,5,6,7,8,9,10], and carbon nanofibers [3,11,12], which are already exploited for various applications such as electronic devices [13,14], sensors [15], renewable energy storage devices [16,17,18], catalyst supporters [19,20], etc. As the applications mainly rely on the capability of successful nano-tailoring of the number of atomic layers deposited in a specific orientation, a reproducible method for the large-scale synthesis is one of the major obstacles for the mass production of OCNs. Conventional micromechanical exfoliation, epitaxial synthesis, and growth methods can produce thin-layered nanostructures, but cannot be implemented for large-scale production [21]. To overcome this issue, plasma-assisted techniques are considered as a promising technique for the large-scale synthesis of CNs, which allows control over their structure and morphology [22,23]. During a plasma-deposition process, precursor gas molecules are dissociated in the plasma and deposited onto the substrate in solid-state. Thus far, various CNs have been synthesized by different PECVD techniques using microwave plasma [6,24], radio frequency inductively coupled plasma (rf-ICP) [25,26], and rf-capacitively coupled plasma (rf-CCP) assisted by H radical injection [27,28,29]. However, the use of flammable gases, such as methane, and the need for external substrate heating during the plasma deposition are the main challenges during the plasma-enabled synthesis for obtaining reproducible results.

Inspired by the recent studies on thermal annealing of natural precursors to obtain CNs, several investigations are reported on the alternative plasma-assisted single-step synthesis of OCNs from environmentally-friendly precursors, such as coconut oil [30], honey [31], butter [32], honeycomb [33], etc. Apart from these precursors, phenol derived xerogels and aerogels are also considered as the precursor sources for the preparation of porous carbon nanoparticles and nanostructures with large surface area [34,35]. Among all varieties of phenol-based polymer gels, resorcinol-formaldehyde (RF) gel is considered as a favorable precursor for the synthesis of open-cell foam-like structured CNs [36] due to their cross-linked polymer network and thermosetting properties [37]. Additionally, it has been reported that RF gel-based structures can be graphitized by catalytic activity upon thermal annealing [38,39,40,41]. However, thermal annealing of phenol-derived gels for the synthesis of CNs is limited due to the requirement of high temperature (900–1800 °C) and lower controllability on the final structure. As plasma surface-treatment has already been used for the orientation-controlled synthesis of the CNs (horizontal to vertical) compared to the thermal annealing methods [32,33], we have explored the possibilities of plasma-assisted techniques for the surface modification of RF xerogels to synthesize OCNs. Modification of the amorphous RF gel to crystalline OCNs (so-called graphitization) by the plasma treatment can alter the morphology, exposed surface area, and functionality, as well as the electrical conductivity of the material. Moreover, the optimized vertical alignment of such CNs contributes to the relatively larger exposure of the active surface compared to the initial arbitrary orientation. Plasma-assisted modification of the carbon species or plasma-deposited CNs are usually formed with a large number of reactive surface sites generated either by the reactive surface carbon atoms or by the surface defects, which can be useful for various applications. Additionally, exploiting the applications of these structures for the detection of commonly used gases can be a new approach for the material-efficient fabrication of OCN based gas-sensors [42].

We demonstrated an environmentally-friendly fast and facile synthesis of OCNs by the plasma-assisted structural reformation of RF xerogel cast on a glass substrate. An rf-ICP ignited by a mixture of Ar and H_2_ gases was used for the surface treatment. Additionally, the proposed synthesis method did not require external substrate heating to produce OCNs. The systematic investigation on the influence of plasma discharge parameters and treatment time on the structural reformation of CNs enabled us to find the optimum conditions for synthesizing OCNs. Structural graphitization, defect generation, and improvement in the orientation of OCNs by plasma surface treatment were confirmed by various surface analytical techniques. The obtained results are used to explain a possible mechanism of the structural reformation of RF gel to graphitized OCNs using an energy-efficient plasma surface treatment technique. The defect-rich properties of CNs reveal the functionality for gas sensing applications potentially at low-temperatures (20–80 °C).

## 2. Materials and Methods

### 2.1. Synthesis of RF Xerogel

All chemicals used in the present study were obtained from Sigma-Aldrich (Merck KGaA, Darmstadt, Germany). RF aqua gel was prepared by dissolving resorcinol (99% purity), formaldehyde (37.6%; methanol stabilized), and sodium bicarbonate (Na_2_CO_3_) in distilled water. A typical gel formulation contained 0.29 M resorcinol, 0.57 M formaldehyde, and 4.0 mM sodium carbonate as the catalyst. The RF mixture was transferred into a 20 mL glass vials and sealed. The glass vials were then kept in the oven at 85 ± 3 °C for 24–36 h. The gelation could occur in approximately 30 h. The solution progressively changed color from clear to yellow, then to orange, and deep red as a function of the reaction time during the gelation process (Figure S1). Prepared RF gels were cast on a borosilicate glass substrate using a doctor blade with a thickness of approximately 100 µm and dried at room temperature to form RF xerogel.

### 2.2. Plasma-Assisted Synthesis of OCNs

Plasma surface treatment on the RF gel was conducted in an rf (13.56 MHz) inductively coupled plasma system that consists of an 80 cm long glass tube with a diameter of 4 cm and a wall thickness of 3 mm. The rf generator was inductively coupled to the system by using a nine-turn water-cooled copper coil. The RF gel was cast on a glass substrate and placed in the glass tube in the center of the coil. Argon and hydrogen gases were used for the discharge. The chamber was pumped down to 3 Pa using a rotary pump before the experiments, and the pressure was increased up to 55 Pa during the experiments. Based on our preliminary experiments, the influence of plasma parameters on the surface deformation was investigated in detail by varying one plasma parameter while keeping other parameters constant. In this study, we investigated the influence of plasma power, gas flow rate, and treatment time; the experimental conditions used in the experiment are listed in Table 1. There was no external heating source used during the plasma treatment, and the substrate temperature was below 300 °C. The schematic diagram of the experimental setup for the plasma-assisted synthesis of the CNs is presented in Figure S2.

### 2.3. Gas Sensing Measurement

Prepared OCNs were peeled off from the glass substrate and placed on a wet polyurethane membrane (GSM 3 g/m^2^, Pardam Nanotechnology, Roudnice nad Labem, Czech Republic) and vacuum filtered to make good adhesion between the membrane and CNs. The sensing property of the OCNs was investigated by measuring the change in sensor resistance using a home-built sensing device, as reported earlier [43]. For preparing the sensor, the OCNs filtered on PU membrane was cut into a dimension of 1.5 cm × 1.5 cm placed on a sensing chip with conductive contacts and connected to the source meter. The resistance was measured by using a Keithley 2460 (Tektronix Inc., Beaverton, OR, USA) high current source-meter at a constant input current of 10 mA, and the data were evaluated with the help of a computer. Two identical 250 mL conical flasks were used as the sensing chamber during the experiment, and ethanol was used as the sensing gas. Ethanol was transferred to one flask, and the other flask was filled with the ambient air. During the sensing measurement, the sensor was manually moved from the “empty” flask to the flask with ethanol (gas on phase), and the sensor was put in the saturated vapor above the ethanol surface. Later, the sensor was put back in the empty flask (gas off phase). The schematic of the experimental setup for the sensor measurements is presented in Figure S3. The time for sensing and recovery process during the gas on and gas off phase was assigned as 180 s. The flasks were heated simultaneously to different temperatures (20, 40, 60, and 80 ℃) using an electric hotplate stirrer and measured the sensing capability towards different ethanol concentrations. The response of the sensor was calculated from:

(1) $$Sensor\;response=\frac{R_{g}-R_{a}}{R_{a}}$$

where *R_g_* is the resistance of a sensor after inserting ethanol, and *R_a_* is the resistance of the sensor in air. The concentration of ethanol vapors at each temperature was calculated using Dalton’s law of partial pressure, and the vapor pressure at different temperatures is given in Supplementary information file, Table S1.

## 3. Characterization Techniques

The surface morphology of the plasma reformed OCNs was analyzed by scanning electron microscope (SEM, JSM 7600F, JEOL Ltd., Tokyo, Japan). The phase composition and the crystal structure of the samples were investigated by the transmission electron microscope, operating at 200 kV (TEM, JEM-2100, Jeol Ltd., Tokyo, Japan). Raman spectra were recorded at various points of the samples to study the structural changes of the nanostructures after plasma treatment using NTEGRA confocal Raman spectrometer (NT-MDT SI, Moscow, Russia) at an excitation wavelength of 633 nm with an incident power ~3 mW at a spot size of 50 µm. The alteration of the crystallinity of the products was assessed by X-ray powder diffractometer (XRD, Philips Analytical PW 3050/60 X’Pert PRO, PANalytical, Almelo, The Netherland) employing Kα irradiation and operating at 45 kV and 40 mA, using incident beam monochromator, while spectra were recorded with a step size of 0.001° in characteristic range of 10–30°. The X-ray photoelectron spectroscopy (XPS PHI-TFA XPS spectrometer, Physical Electronics Inc., Chanhassen, MN, USA) analysis was employed to evaluate the surface composition and chemical bonding of the nanostructures using an Al-monochromatic X-ray source at the energy of 1486.6 eV. The changes in the functional entities of the RF xerogel after the plasma surface treatment were analyzed by Fourier transform infrared spectroscopy (FTIR). FTIR spectrum was recorded by GX FTIR spectrometer (Perkin Elmer Inc., Waltham, MA, USA) equipped with a photoacoustic detector. The spectra were obtained over wavelengths between 4000 and 500 cm^−1^ (mid-infrared region) with 8 cm^–1^ resolution by averaging 64 scans.

## 4. Results and Discussions

**Figure 1:**
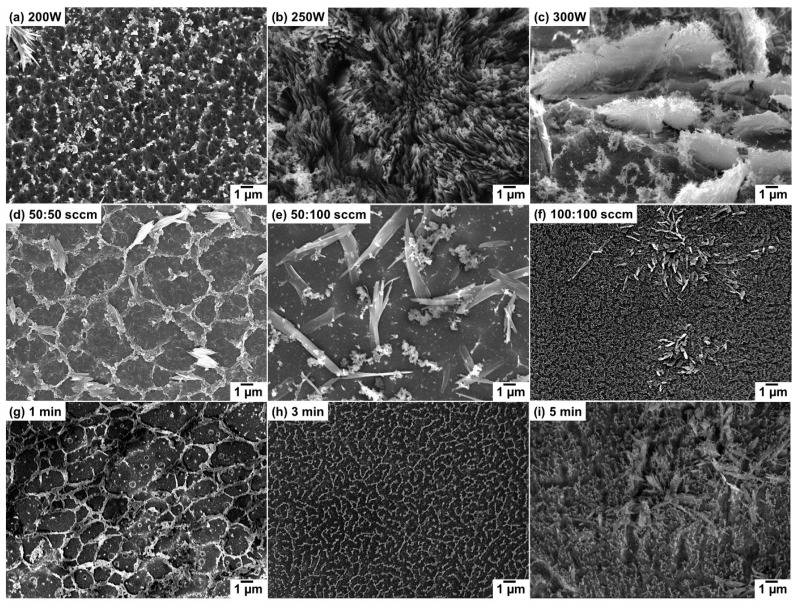
Secondary electrons (SE) SEM micrographs of carbon structures after plasma surface treatment on RF gel at different treatment conditions: (**a**–**c**) different power at 100:50 sccm and 8 min; (**d**–**f**) different flowrates at 250 W and 8 min and (**g**–**i**) different time at 250 W and 100:50 sccm.

To understand the effect of plasma treatment on the RF gel morphology, a series of CNs were synthesized at different plasma conditions. The first parameter varied during the experiment was plasma power (200–300 W), while keeping flow rate (100:50 sccm) and growth time (8 min) constant. During the plasma surface treatment of RF gel cast on a glass substrate, the first observation is a noticeable change of color from deep red to black, which can be due to the changes in structure and morphology of precursor. Thus, the change in surface morphology of the RF gel after each plasma treatment was analyzed by SEM, and the effect of plasma power on the surface morphology of CNs is presented in Figure 1a–c. The morphological characteristics of CNs with respect to each plasma power suggests that the structure is changing with the increase in power. At a power of 200 W, a microscale flat film with randomly distributed fibrils is formed on the substrate (Figure 1a). At a power of 250 W, densely-packed vertically oriented thick flakes, with a thickness of ~80–100 nm, are observed (Figure 1b). At a further increase of power to 300 W, a free-standing individual flake-like structure is observed (Figure 1c). The CNs are damaged when using the power of 300 W, and while comparing CN growth at powers of 150 and 250 W, it is observed that the growth is more significant in the latter. Consequently, the power of 250 W was taken as an optimum plasma power for investigation of the influence of gas flow rate and treatment time on CN growth. The flow rate of the gas mixture is also considered as a key factor on the surface reformation. Thus, the plasma treatment was conducted at different flow rates (pressure) of Ar: H_2_ as 50:50 sccm (31 Pa), 50:100 sccm (41 Pa), and 100:100 sccm (53 Pa) at a constant plasma power (250 W) and treatment time of 8 min. A microporous structure is observed at a gas mixture ratio of 50:50 sccm (Figure 1d). When the flow ratio is 50:100 sccm, randomly distributed thick fiber-like structures are formed on the substrate (Figure 1e). A further change in the flow ratio to 100:100 sccm leads to the formation of dense CNs with randomly distributed nanofibers (Figure 1f). Evaluation of the influence of flowrate also confirms that densely packed OCNs are growing from RF gel after 8 min of surface plasma treatment at a plasma power 250 W and gas flow ratio 100:50 sccm.

**Figure 2:**
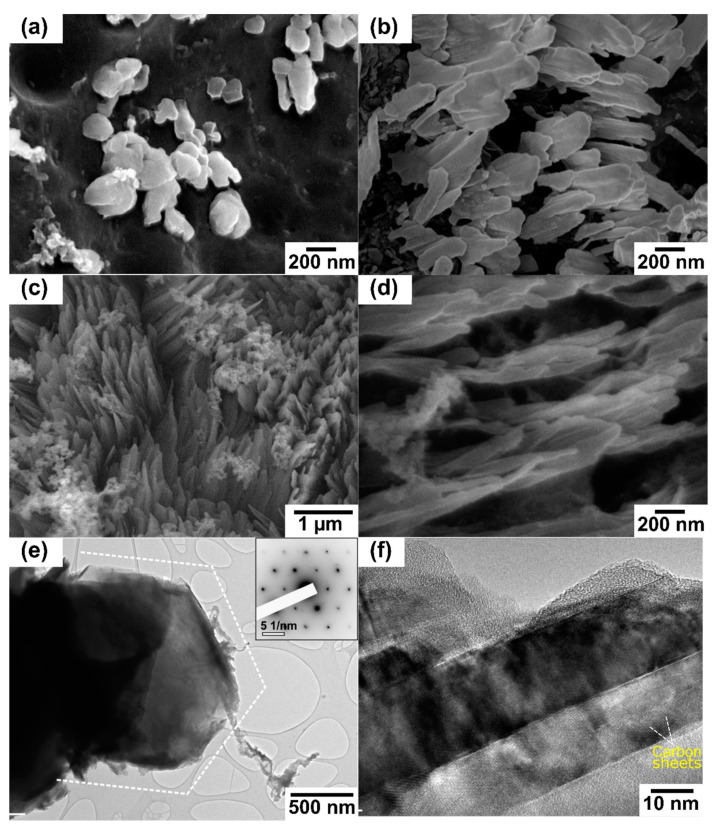
High-resolution SEM micrographs of the carbon nanostructure formation during the time-dependent growth at 250 W and 100:50 sccm after: (**a**) 3 min; (**b**) 5 min; (**c**) and (**d**) 8 min treatment; (**e**) overview TEM micrograph of individual graphite-like hexagonal flake (diffraction in inset), and (**f**) side-view of graphite-like flake, showing several stacks of well-crystalline carbon structures.

Thus, to investigate the formation of OCNs by plasma processing, time-dependent plasma treatment was performed. The progress of morphology was investigated at different treatment times, 1, 3, 5, and 8 min, as presented in Figure 1b,g,h,i, respectively. The morphology of RF gel treated for 1 min resembles an interconnected ring-like structure with large interspacing. After 3 min of plasma treatment, the surface is filled with a broken ring-like structure (Figure 1h). Further increase of treatment time to 5 min leads to the formation of the combined ring and fiber-like structures on the substrate, where the fiber-like structures seeded on the surface are also seen after 3 min treatment. The longest plasma treatment of 8 min resulted in the removal of ring-like structures and the formation of densely packed OCNs (Figure 1b). Additionally, further plasma treatment longer than 8 min has resulted in the erosion of all of the precursors from the substrate. High-resolution micrographs of the time-dependent growth of CN structures during the plasma treatment process are presented in Figure 2a–d. The nucleation of the CN structures started after 3 min treatment, and the growth is facilitated with time. The CN structures after 8 min treatment are in the form of vertically orientated flake-like structures (Figure 2c,d).

The phase composition and crystal structure of CN structures formed after 8 min plasma surface treatment was further investigated by TEM. The morphology of individual flake-like structure has a characteristic hexagonal outline, as seen in Figure 2e. The selected area electron diffraction pattern (Figure 2e, inset) corresponds to the graphite viewed down the [0001] zone axis (c-axis), showing {10-10} and {1-100} reflections. When looked at from the side (down the a- or b- axis), at sufficiently high magnification, we can resolve individual highly-crystalline layers separated for about 3.4 Å, bonded together by Van der Waals bonds, as seen in Figure 2f. The thickness of such stacks is several tens of nanometers. Therefore, plasma surface treatment for 8 min at a power 250 W and Ar: H_2_ flowrate 100:50 sccm was considered as the optimum condition for the synthesis of OCNs. Additionally, the change in chemical composition and improvement in structural quality during the time-dependent growth of OCNs were investigated to identify the characteristics of the deposited OCNs.

**Figure 3:**
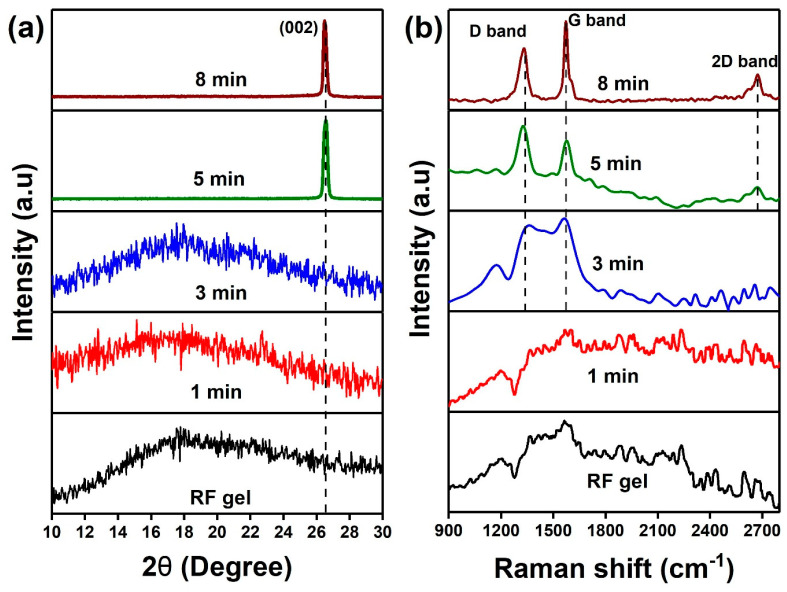
(**a**) XRD spectra and (**b**) Raman spectra of CNs obtained after the time-dependent plasma surface treatment on RF gel at 250 W and flow rate 100:50 sccm.

The evolution of the crystal structure of the plasma-treated samples was assessed by XRD (Figure 3a). The results show there are no clear diffraction peaks observed in 1 min and 3 min plasma-treated RF gel, and in the diffraction spectra, only broad and diffused scattered reflections can be observed, characteristic for amorphous or poorly-crystalline structures with only short-range ordering. In contrast, the samples that were treated 5 and 8 min exhibit sharp diffraction peak at 2θ = 26° corresponding to (0 0 2) lattice plane of graphite [38], which is typical for so-called graphitic structures where we have at least few graphene-like monolayers stacked in c-direction, resembling the graphite structure.

The chemical composition of the CNs produced during the time-dependent structural reformation of RF gel by plasma treatment was analyzed with surface analytical techniques. Raman spectra were used to investigate the structural organization of the samples (Figure 3b). A broad peak at 1350–1600 cm^−^^1^ region is observed for the non-treated RF gel, and similar characteristics are observed in the sample after 1 min of plasma treatment. The peak can be ascribed to the presence of aromatic rings present in the polymer chain [44]. On the other hand, 3 min treated samples exhibit two broad peaks at 1362 and 1570 cm^–1^ characteristic for the amorphous carbon species, while 5 and 8 min plasma-treated samples display an evolution of well-distinguished peaks at 1330, 1581 and 2700 cm^–1^ that correspond to characteristic D, G and 2D peaks in defected graphitic carbon structures [45,46,47]. Detailed information on the peak positions is presented in Table S2. The results obtained from Raman and XRD analysis are confirming the graphitization of CNs after 5 and 8 min plasma treatment.

**Figure 4:**
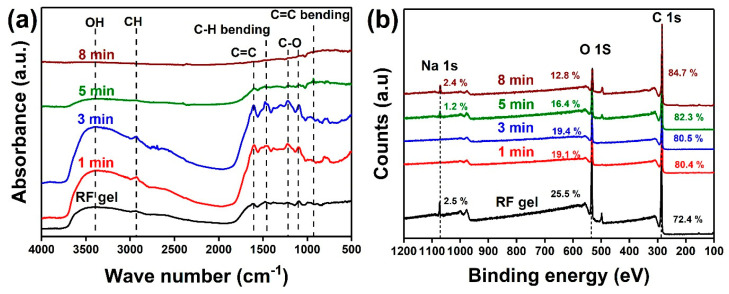
(**a**) FTIR spectra and (**b**) XPS survey spectra of OCNs obtained after the time-dependent plasma surface treatment on RF gel at a power:250 W, and flow rate:100:50 sccm.

The changes in the functional entities upon the plasma treatment were further investigated by the FTIR analysis (Figure 4a). FTIR spectra of non-treated RF gel and 1–5 min treated samples indicates peaks at 3200–3410 cm^−1^, 1620 cm^−1^, 1460 cm^−1^, and 1300 cm^−1^ corresponding to the vibrations from the hydroxyl group, aromatic group, CH_2_ group, and alkyl-phenyl ether groups respectively [48,49,50]. Compared to this, all the chemical bond vibrations were diminished in the FTIR spectra of the OCNs formed after 8 min plasma treatment, which is confirming the graphitic characteristic of the OCNs [51]. This result suggests that the oxygen-containing functional groups are eliminated from the RF surface during the CNs formation.

**Figure 5:**
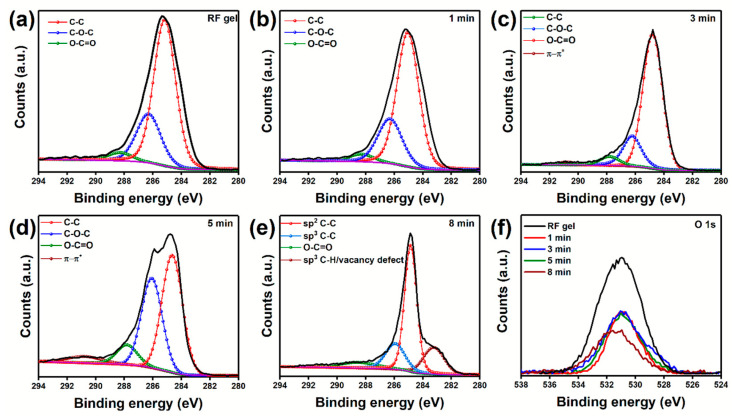
High-resolution C 1s spectra of RF gel and CNs obtained after the time-dependent plasma surface treatment at a power of 250 W and flow rate of 100:50 sccm: (**a**) RF gel (**b**) 1 min; (**c**) 3 min; (**d**) 5 min; (**e**) 8 min; and (**f**) O 1s spectra of RF gel and CNs after each stage of plasma surface treatment.

To confirm the changes in functional entities during the CN formation, changes in the chemical composition of RF gel during each stage of time-dependent plasma treatment were studied by XPS analysis. The loss in the atomic concentration of the oxygen during the conversion of RF gel to OCNs is evident in Figure 4b. Interestingly, the peak observed for metallic Na is diminished after 1 and 3 min of plasma treatment. However, the peak from Na is observed in 5, and 8 min plasma treated samples, which can be due to the recombination of the functional entities on the surface during the reformation. The high-resolution C 1s spectra of the non-treated and 1, 3 and 5 min treated samples were deconvoluted to three main peaks located at 284.8, 286.3 and 288.3 eV, corresponding to C–C bonds, sp^3^ C–C/carbon singly-bound to oxygen, and the carbon in carbonyl groups (Figure 5a–e) [52]. In addition to these peaks, the plasma-treated sample exhibited an additional peak at 290.8 eV, corresponding to the π-π* shake-up satellite. Moreover, the area of C–O–C component after 5 min treatment is higher than the other samples, indicating that the initial CNs formation is occurred by the structural reformation of the C–O–C bonding and forming sp^3^ C–C. Further plasma treatment enhances the removal of these sp^3^ C–C components and improve the sp^2^ C–C characteristics of the CNs and creates vacancy defects in the CNs, which is evident in the C 1s spectra of the 8 min plasma-treated sample. Detailed information on the deconvolution of the peaks, peak position, full width half maximum, and peak area are described in the Supplementary information file, Table S3. The reduction in the peak intensity of O 1s spectra after the plasma treatment also confirms the removal of oxygen functional groups upon the plasma treatment (Figure 5f). The changes observed in O 1s and C 1s spectra and evolution of a peak in the C 1s spectra corresponds to vacancy defect confirming the plasma-enabled formation of defect-rich graphitic OCNs from RF gel.

**Figure 6:**
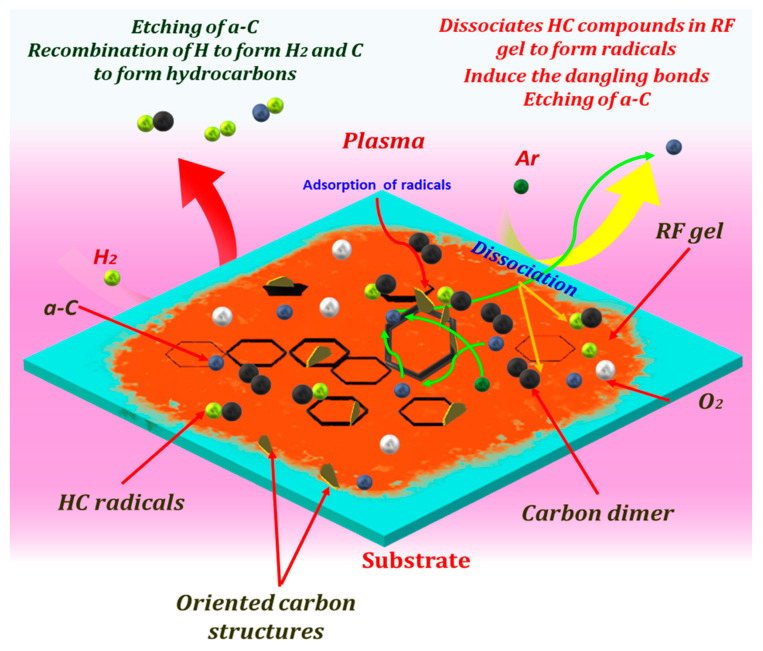
Schematic of the possible mechanism of the growth of OCNs by the plasma reformation of RF gel at the plasma discharge conditions power of 250 W, time of 8 min, and the flow rate of 100:50 sccm.

In thermal annealing methods, a substrate temperature above 600 °C is the key factor for the graphitization, and it could only decompose the carbon-containing precursors into carbonaceous building blocks [53]. In this study, the RF gel cast on the glass substrate directly interacted with the plasma generated species, and the substrate temperature during the process are comparatively lower than the thermal annealing methods, suggesting that temperature was not likely the determining factor of the transformation of the sample in plasma. Thus, the synthesis of OCNs could be explained as the plasma-enhanced etching and constructive interplay between the discharge gases. During the plasma surface treatment, argon and hydrogen are dissociated and form various species in plasma (Figure S4). H_2_ is considered as a critical factor in the nucleation and growth of many carbon-based nanostructures [1,54], while Ar is considered as the amorphous etchant. As seen from the time-dependent changes on the surface morphology (Figure 2), at the initial stage, carbon building blocks are extracted from the top-layer of RF gel due to the ion bombardment and neutral radical interaction and re-form into hexagonal carbon rings, which is considered as the essential elements for the growth of OCNs [55]. The presence of reactive H atoms allows the diffusion of these building units on the surface through the recombination-mediated energy dissipation and facilitating the nucleation of CNs [32,56]. Further treatment is enhancing the extraction from these structures as well as seeds the nanostructures, and finally, CNs were formed by the continuous recombination of building units. These diffusions of building units and recombination are influenced by the different plasma discharge parameters and resulted in the various morphology of such obtained nanostructures. Based on the experimental observations, a possible mechanism of the plasma-enhanced surface reformation of RF gel to OCNs is illustrated in Figure 6.

The graphitization of the carbon is enhanced by the removal of impurities and to form a continuous sp^2^ carbon network by the H atoms present in plasma. However, the etching rate becomes dominant at higher power and gas flow rates, which can create more defects and structural damage [57]. On the other hand, insufficient etching and recombination at a low concentration of gases and low plasma power have resulted in the amorphous carbonaceous structures.

## 5. Gas Sensor Properties

**Figure 7:**
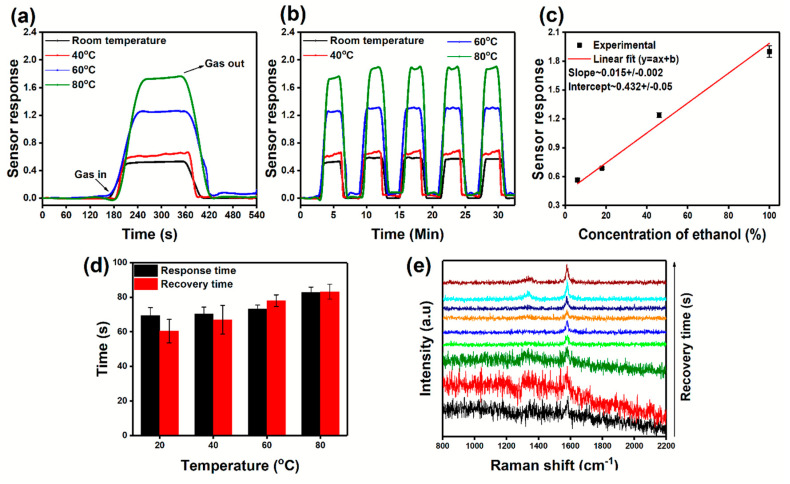
Gas sensor properties of OCNs prepared from RF gel: (**a**) sensor response to ethanol vapor at different heating temperatures, (**b**) repeatability of sensor response towards ethanol vapor; (**c**) sensor response towards the ethanol concentration as a function of heating temperature; (**d**) response and recovery time of OCN-based sensors towards ethanol at different heating temperature; and (**e**) ex-situ Raman spectra recorded on OCN sensor during the recovery of ethanol after sensor response at an acquisition time of 4 s with an interval of 6 s.

Vertically oriented morphology and defect-rich graphitic characteristics of the CNs can enhance the adsorption of chemical species and change the electrical properties during the process. Thus, the OCNs obtained after 8 min of surface treatment at a power of 250 W and gas flow rate of 100:50 sccm were used for the gas sensing measurements. During the sensing measurement, the sensor was kept in an empty flask for 180 s and then placed into the ethanol environment for 180 s to detect the ethanol vapor. The sensor responses at room temperature and different heating temperatures are presented in Figure 7a, which indicates the increase in sensor response with the increase in ethanol vapor concentration. It has been seen that the OCNs significantly react with ethanol, even at room temperature, with a response value of 0.56 ± 0.0.2. The response was increased to a value of 1.86 ± 0.06 at a heating temperature of 80 °C, about three times higher than at room temperature. It is worth noting that the performance was repeatable and indicating the sensor did not lose the sensing capabilities after the initial response cycle (Figure 7b).

The sensor response towards the increase in ethanol concentration as a function of the heating temperature is presented in Figure 7c. The OCN-based sensor exhibits a linear increase in the response towards ethanol vapor with an increase in concentration. The linear fitting denoted as:

Sensor response *S*= 0.432 + (0.01 × *Concentration of ethanol*) (2)

A comparison between the response and recovery times of the sensor at room temperature and the different heating temperatures is represented in Figure 7d. The response time *T_res_* was estimated as the time taken to achieve 90% of the maximum change in resistance. In contrast, recovery time *T_rec_* was calculated as the time taken to recover down to 90% of the maximum resistance change. It is seen that the response and recovery times are increasing with the heating temperature. The sensor took 69 s for the initial response at room temperature, while it increased to 83 s at 80 °C. The sensor attains the initial resistance within 60 s at room temperature after the sensing cycle. On the other hand, the sensor took 84 s for the complete recovery after the sensing cycle at a heating temperature of 80 °C. Additionally, there is a slight change in baseline resistance observed after the first sensing cycle at heating temperatures of 60 and 80 °C. During the sensing cycle, ethanol molecules replace the oxygen on the surface, and during the recovery process, oxygen molecules are partly occupied at the surface and resulted in the change in baseline resistance and prolonged response and recovery time [58,59].

The repeatability of the sensing cycle, indicating that the sensor is not losing the sensing capabilities after the first sensing cycle, can be explained by the chemical stability of the sensor. To understand the chemical changes occurring during the sensing cycle, ex-situ Raman spectra were recorded on the OCN-based sensor after the sensor response and are presented in Figure 7e. The spectra were recorded at an acquisition time of 4 s from a single point with an interval of 6 s to understand the chemical changes that occurred after the maximum sensor response. The signal from the graphitic characteristics of OCNs is suppressed during the initial spectra, which could be due to the presence of the ethanol vapors. Later, the well-defined Raman peaks are visible with the increase in recovery time, suggesting that the ethanol is completely desorbed from the surface, and there is no chemical modification takes place during the sensing measurements. These results are indicating that the OCN-based gas sensor shows excellent sensing capability towards ethanol molecules.

## 6. Conclusions

We synthesized graphitized OCNs using an environmentally benign radio-frequency plasma surface-treatment technique. Resorcinol-formaldehyde gel cast on a glass substrate was used as the precursor source for the synthesis of graphitized OCNs. The time-dependent evolution of the OCNs with vertical orientation and influence of plasma discharge parameters on the structure-controlled synthesis was studied in detail. The graphitic characteristics with structural defects were explored using various surface analytical techniques, such as SEM, TEM, XRD, XPS, and Raman analysis. Synthesized defect-rich OCN structures exhibit excellent gas sensing properties to ethanol at room temperature. The response of the sensor towards the increase in the concentration of ethanol was also explored. It is also to be noted that the sensor response, response time, and recovery time of the sensors were increasing with the ethanol concentration. Thus, with further optimization, these OCNs produced from polymer resources could potentially form the backbone of a novel sensing platform based on nanomaterials. Moreover, improving sensitivity and selectivity towards different gases needs to be investigated for the future applications of the OCN-based sensor.

## Figures and Tables

**Table 1:** Experimental conditions for plasma-assisted structure reformation of RF gel to CNs.

Sample	Plasma Power (W)	Flow Rate Ar:H_2_ (sccm)	Growth Time (min)
1	200	100:50	8
2	250	100:50	8
3	300	100:50	8
4	250	50:50	8
5	250	50:100	8
6	250	100:100	8
7	250	100:50	1
8	250	100:50	3
9	250	100:50	5

## Supplementary Materials

The following are available online at https://www.mdpi.com/2079-4991/10/9/1704/s1, Figure S1. The progressive change in color of RF gel as a function of reaction time during the gelation process from clear (initial stage) to deep red (after complete gelation), Figure S2. Experimental setup for the rf-ICP system used for the synthesis of CNs from RF gel by plasma surface treatment, Figure S3. Experimental setup for the home-built sensing device for ethanol detection, where the sensor is placed to the saturated vapor above the liquid ethanol, Figure S4. Optical emission spectra of the Ar/H_2_ plasma ignited at 250 W, and 100:50 sccm flow rate with all the species excited during the surface treatment process, Table S1. The concentration of ethanol at different temperatures calculated using Dalton’s law, Table S2. Details of the peak positions observed in the Raman spectra of CNs obtained after 3, 5 and 8 min of plasma treatment, Table S3. Peak position, FWHM, and roughly estimated concentration of the peak components in XPS C 1S spectra of the time-dependent growth of carbon nanostructures.
