# Supplementary material for: Oriented Carbon Nanostructures from Plasma Reformed Resorcinol-Formaldehyde Polymer Gels for Gas Sensor Applications
Source: Nanomaterials (Basel). 2020 Aug 29;10(9):1704. doi: 10.3390/nano10091704 (PMC7559324; doi:10.3390/nano10091704)
Supplement: Supplementary file 1 [file nanomaterials-10-01704-s001.pdf]

nanomaterials

Supporting information

Oriented Carbon Nanostructures from Plasma Reformed Resorcinol-formaldehyde Polymer Gels for Gas Sensor Applications

Neelakandan M Santhosh [1,2], Aswathy Vasudevan [1,2], Andrea Jurov [1,2,] Gregor Filipič [1], Janez Zavašnik [1], Uroš Cvelbar [1*]

[1] Department of Gaseous Electronics, Jožef Stefan Institute, Jamova cesta 39, SI-1000 Ljubljana, Slovenia, EU
[2] Jožef Stefan International Postgraduate School, Jamova cesta 39, SI-1000 Ljubljana, Slovenia, EU
* Correspondence: uros.cvelbar@ijs.si;

RF gel preparation

Resorcinol-formaldehyde gel was prepared using a simple polymer gelation method, reported elsewhere [1]. 1.28 g of resorcinol, 2 ml of formaldehyde and 0.04 g of sodium carbonate was dissolved in deionized water and kept in an oven at 85 ± 3 °C for 24-36 h. During the gelation period, the solution changes the colour from clear to yellow, then to orange, and deep red as a function of the reaction time. The solution was deep red after the complete gelation, as shown in Figure S1. In the next step, the gel was used for the synthesis of CNs using plasma surface treatment.

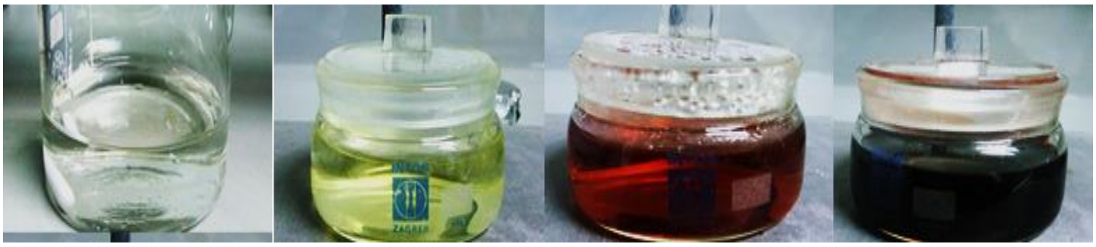

Figure S1. The progressive change in colour of RF gel as a function of reaction time during the gelation process from clear (initial stage) to deep red (after complete gelation).

Experimental set up of the rf-ICP system

The plasma-surface treatment was carried out in a radio-frequency plasma inductively coupled through a 9-turn copper antenna. RF gel cast on a glass substrate was placed in the centre of the coil, and the chamber pumped down to 3 Pa before the experiments. Argon and hydrogen were used as the discharge gases, which were inserted to the chamber from the sides. During the discharge, different flowrates and plasma power were used for surface treatment. Schematic diagram of the rf-ICP system for the plasma-surface treatment is presented in Figure S2.

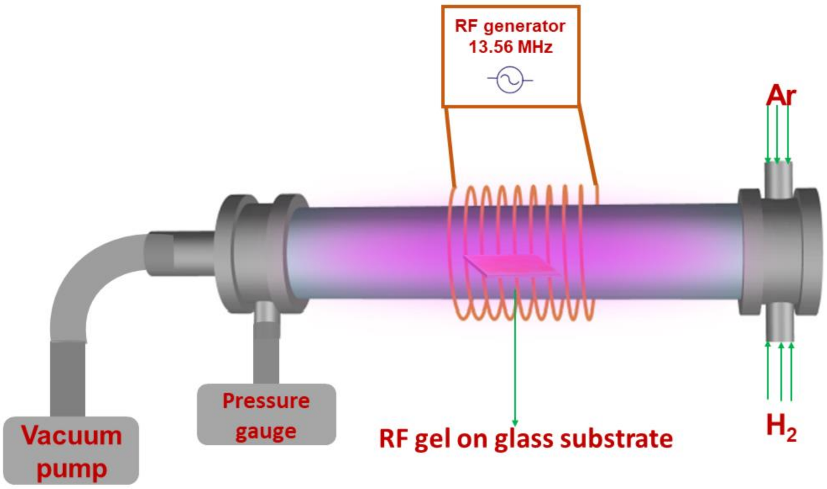

Figure S2. Experimental setup for the rf-ICP system used for the synthesis of CNs from RF gel by plasma surface treatment.

Experimental setup for sensing measurements

The OCN structure filtered on PU membrane was cut into a dimension of 1.5*1.5 cm placed on a sensing chip and connected to the source meter. Two identical 250 ml conical flasks were used as the sensing chamber during the experiment, and ethanol was used as the sensing gas. Ethanol was transferred to one flask, and the other flask was filled with the ambient air. During the sensing measurement, the sensor was moved manually from the "empty" flask to the flask with ethanol (gas on phase) and put the sensor to the saturated vapour above the ethanol surface. Later, the sensor was put back to the empty flask (gas off phase). The schematic of the experimental setup for the sensor measurements is presented in Figure S3.

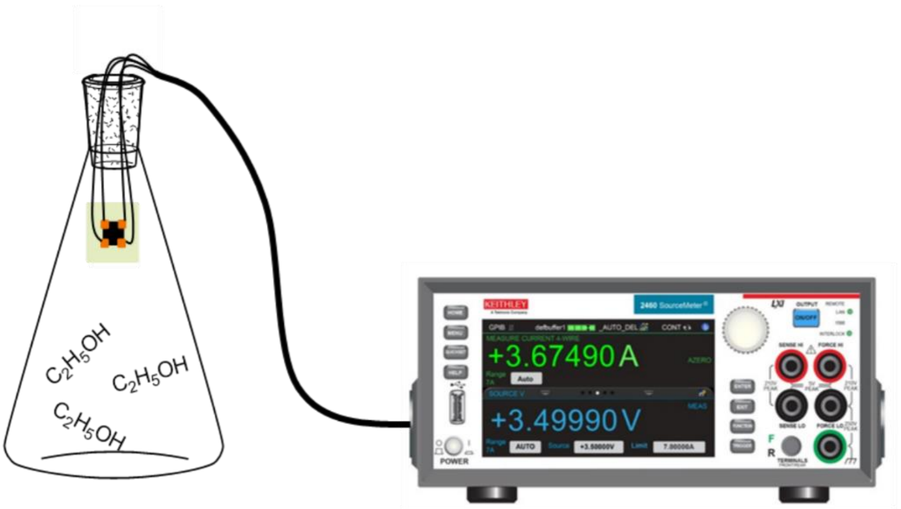

Figure S3. Experimental setup for the home-built sensing device for the ethanol detection, where the sensor placed to the saturated vapor above the liquid ethanol.

Calculation of ethanol concentration

The concentration C_{et} of ethanol inside the sensing, the chamber was controlled by heating the known volume of ethanol to different temperatures. Since the sensing chamber is filled with a mixture of gases (air and ethanol vapour), Dalton's law was used for the estimation of the volume-based concentration of ethanol as following:

$$p_{et} = p_{tot}\, c_{et}$$

where p_{et} is the vapour pressure of ethanol, p_{tot} is the atmospheric pressure (101.325 kPa) and c_{et} is the volume-based concentration of ethanol. The saturated vapour pressure of ethanol was obtained from the publicly available online databank (table footnote [a]). The calculated concentrations of ethanol are presented in Table S1.

Table S1. The concentration of ethanol at different temperature calculated using Dalton's law.

Temperature	Vapour pressure of ethanol (kPa)	Concentration of ethanol (%)
20 °C	5.82625	~5.75
40 °C	17.8258	~17.59
60 °C	46.7509	~46.14
80 °C	108.432	~107

[a] http://ddbonline.ddbst.com/AntoineCalculation/AntoineCalculationCGI.exe?component=Ethanol

Raman analysis

The changes occurred in the structure quality of the CNs during the plasma treatment were evaluated by Raman spectroscopy. The peaks of the Raman spectra observed in the CNs after 3-8 min plasma treatment is showing the characteristic behaviour of the graphitic structure. The peak around 1330 cm^{-1} (D band) is ascribed to the A_{1g} breathing mode of six-atom rings in the 1st Brillouin zone boundary K or K' [2]. The G band observed at ~1581 cm^{-1} is assigned to the E_{2g} symmetry corresponds to the one-phonon Raman scattering process at the 1st Brillouin zone centre [3,4]. Evolution of peak at ~2700 cm^{-1}, corresponds to the second-order of zone boundary phonons and activated by the scattering of two phonons with opposite wave vectors.

Table S2. Details of the peak positions observed in the Raman spectra of CNs obtained after 3, 5 and 8 min of plasma treatment.

Sample condition	Peak 1 (cm^{-1})	Peak 2 (cm^{-1})	Peak 3 (cm^{-1})
3 min	1337	1577	
5 min	1328	1578	2662 (cm^{-1})
8 min	1330	1582	2665 (cm^{-1})

X-ray photoelectron spectroscopy (XPS) analysis

The chemical composition analysis of the prepared CNs was investigated by XPS. The C 1s spectra of the non-treated RF gel was deconvoluted to three main peaks located at 284.8, 286.3 and 288.3 eV, corresponding to C-C bonds, carbon singly-bound to oxygen, and the carbon in carbonyl groups [5]. C 1s spectra of the 1 min, 3 min and 5 min treated shows similar peak characteristics and exhibits an additional peak at 290.8 eV corresponding to the π-π* shake-up satellite. Addition to this, the OCNs formed after the 8 min plasma treatment the peak observed at 283.2 eV corresponds to the sp^3 C-H/ vacancy defect and hence confirms the defected nature of CNs [6]. Compared to remaining samples, the peak observed at 284.8 eV is narrow with a full-width half-maximum of 1 eV, confirms the graphitic behaviour of the carbon nanostructures. Detailed information on the peak position and roughly estimated composition ratio (%) of the deconvoluted peaks of the high-resolution spectra are summarized in Table S1.

Table S3. Peak position, FWHM, and roughly estimated concentration of the peak components in XPS C 1S spectra of the time-dependent growth of carbon nanostructures.

Sample	Peak 1			Peak 2			Peak 3			Peak 4		
	Position (eV)	FWHM (eV)	Area %	Position (eV)	FWHM (eV)	Area %	Position (eV)	FWHM (eV)	Area %	Position (eV)	FWHM (eV)	Area %
RF gel	284.8	1.7	66.3	286.3	2.0	22.7	288.3	2.0	11.0			
1 min	284.8	1.7	69.5	286.3	2.0	26.1	288.3	2.0	4.4			
3 min	284.8	1.6	74.4	286.3	1.5	18.5	288.1	1.8	5.3	290.8	1.5	1.6
5 min	284.8	1.6	48.9	286.1	1.5	37.3	287.9	1.8	9.0	290.8	2.1	4.7
8 min	284.8	1.0	56.0	285.9	1.5	18.4	288.4	2.2	7.5	283.2	1.7	18.7

The optical emission spectra (OES) analysis

The optical emission spectra (OES, broad range spectrometer LR1, ASEQ) was recorded every 30s during the plasma treatment of RF gel to observe the excited species during the process of CNs formation. The spectra were recorded for 8 min at plasma power 250 W and Ar: H_2 flowrate 100:50 sccm. In the beginning, only noise can be seen in the spectrum, which could be explained with plasma species going into fast chemical reactions with the RF gel so that the recombination of excited species is too fast to record with the used spectrometer. After 2 min we observed a formation of OH peak at 309 nm and H_α at 656 nm. After another minute, other species start to show – N and Ar lines become distinguishable along with the nitrogen continuum that is always observed inside low-pressure rf plasma. Even though Ar was a working gas, we observe OH in the spectrum; it can be because of the excitation of the top layer of RF gel during the surface reformation.

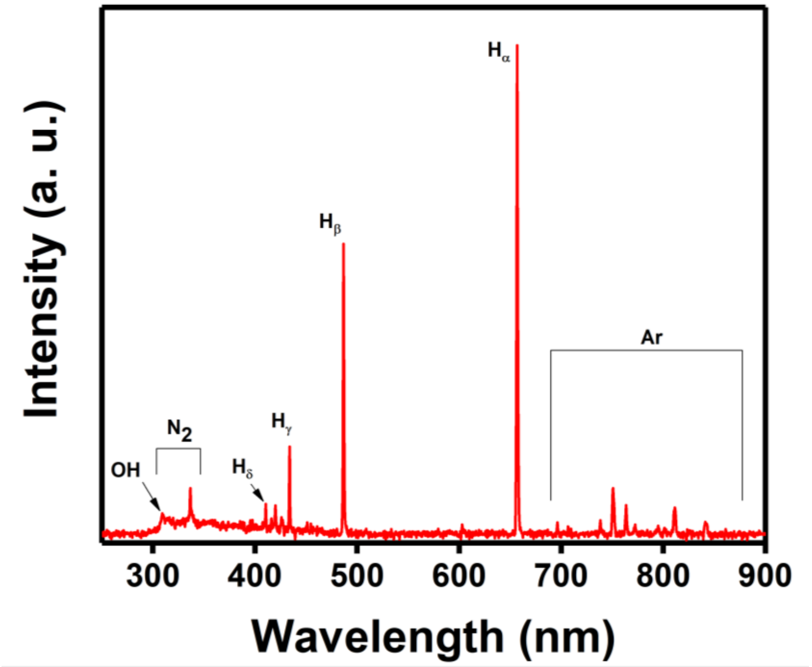

Figure S4. Optical emission spectra of the Ar/H_2 plasma ignited at 250 W, and 100:50 sccm flow rate with all the species excited during the surface treatment process.

References

1. Pekala, R. W. Organic aerogels from the polycondensation of resorcinol with formaldehyde. *J. Mater. Sci.* **1989**, doi:10.1007/BF01139044.
2. Ferrari, A. C.; Basko, D. M. Raman spectroscopy as a versatile tool for studying the properties of graphene. *Nat. Nanotechnol.* **2013**, *8*, 235–246, doi:10.1038/nnano.2013.46.
3. Maslova, O. A.; Ammar, M. R.; Guimbretière, G.; Rouzaud, J. N.; Simon, P. Determination of crystallite size in polished graphitized carbon by Raman spectroscopy. *Phys. Rev. B - Condens. Matter Mater. Phys.* **2012**, doi:10.1103/PhysRevB.86.134205.
4. Ammar, M. R.; Charon, E.; Rouzaud, J. N.; Aleon, J.; Guimbretière, G.; Simon, P. On a reliable structural characterization of polished carbons in meteorites by Raman microspectroscopy. In *Spectroscopy Letters*; 2011.
5. M. Santhosh, N.; Filipič, G.; Kovacevic, E.; Jagodar, A.; Berndt, J.; Strunskus, T.; Kondo, H.; Hori, M.; Tatarova, E.; Cvelbar, U. N-Graphene Nanowalls via Plasma Nitrogen Incorporation and Substitution: The Experimental Evidence. *Nano-Micro Lett.* **2020**, *12*, 53, doi:10.1007/s40820-020-0395-5.
6. Fujimoto, A.; Yamada, Y.; Koinuma, M.; Sato, S. Origins of sp3C peaks in C1s X-ray Photoelectron Spectra of Carbon Materials. *Anal. Chem.* **2016**, doi:10.1021/acs.analchem.6b01327.
